# The complement system contributes to the immunosuppressive microenvironment of uveal melanoma

**DOI:** 10.1186/s12967-026-07910-y

**Published:** 2026-03-09

**Authors:** Iryna Zherka, Helen Kalirai, Dominika Majorova, Sarah E. Coupland, Monica M. Olcina

**Affiliations:** 1 Department of Oncology, University of Oxford, Old Road Campus Research Building, Roosevelt Drive, Oxford, OX3 7DQ UK; 2 Liverpool Ocular Oncology Research Group, University of Liverpool, Liverpool, UK

**Keywords:** Uveal melanoma, Complement system, Single-cell RNA-sequencing, Immunosuppressive tumor microenvironment, Drug repurposing

## Abstract

**Background:**

Uveal melanoma (UM) is a rare disease, but the most common intraocular malignancy in adults, with incidence rates in Europe ranging from 1.3 to 8.6 cases per million annually. Although local tumor control is often effective, up to 50% of patients develop systemic metastases for which treatment options remain limited. Complement gene expression has been linked to poor prognosis in UM, but its role in tumor biology is still not well understood.

**Methods:**

Hypothesizing that dysregulated complement signaling fosters an immunosuppressive tumor microenvironment and promotes progression, this study for the first time investigated the role of the complement system in primary and metastatic UM. We applied an integrated approach utilizing publicly available UM bulk, single-cell RNA sequencing and proteomic data as well as immunohistochemistry of human samples, and RNA expression analysis. We also compared complement associated protein expression between primary UM (pUM) and normal ocular tissue, as well as metastatic UM (mUM) and normal liver.

**Results:**

pUM preferentially expressed early complement components over terminal elements, with early factors demonstrating prognostic significance. High-risk tumors showed elevated secretion of C1S and C1R proteases. Fibroblasts and macrophages constituted the main sources of complement genes. mUM exhibited an expanded role for C3 relative to primary tumors. Fibroblasts were predicted to drive formation of an immunosuppressive microenvironment via RPS19–C5AR1, C3–ITGAM, and C3–C3AR interactions with macrophages and myeloid-derived suppressor cells. Trajectory analysis identified the iCAF phenotype as a transitional state between normal liver fibroblasts and mCAF, suggesting fibroblast education and involvement in pre-metastatic niche formation.

**Conclusion:**

Our findings identified C1S and C3 complement components as promising therapeutic targets. The availability of clinically approved complement inhibitors supports potential repurposing for high-risk pUM and mUM, offering a novel strategy to improve treatment outcomes.

**Supplementary Information:**

The online version contains supplementary material available at 10.1186/s12967-026-07910-y.

## Introduction

Uveal melanoma (UM) is a rare disease but the most common intraocular malignancy in adults, arising from melanocytes located in the uveal tract of the eye. Despite effective primary tumor management, approximately 50% of patients develop systemic metastases. While less extensively studied than primary disease, metastatic UM (mUM) accounts for most of the UM-related mortality with an overall survival (OS) of less than 12 months [1]. mUM is lacking effective treatment options, and the National Comprehensive Cancer Network (NCCN) guidelines still recommend clinical trials as a first line treatment [2]. Liver-directed therapies, such as resection, intraarterial liver chemotherapy or transarterial chemoembolization, provide a certain level of disease control in oligometastatic disease [1, 3]. Unlike cutaneous melanoma, immune checkpoint blockade shows limited activity in mUM; in a Phase 2 trial, nivolumab plus ipilimumab achieved a median OS of 12.7 months in treatment-naïve patients [4]. Tebentafusp, a bispecific T-cell–redirecting protein targeting gp100, improves OS by 5.7 months over immunotherapy or chemotherapy in a Phase 3 trial [5]. It was approved for mUM treatment and needs HLA testing with approximately 50% of patients being eligible.

UM is generally considered immunologically “cold,” with a suppressive immune microenvironment. Moreover, UM displays a unique immunological paradox: unlike most cancers, higher number of tumor-infiltrating immune cells are associated with poor prognosis [6, 7]. Considering current knowledge, potential UM-specific therapeutic strategies include reprogramming the immunosuppressive tumor microenvironment or leveraging the innate immune system.

Being an essential part of innate immunity, the complement system has been demonstrated to shape the tumor immune microenvironment, thereby influencing tumor prognosis [8]. C3a and C5a were shown to remodel the tumor microenvironment via multiple context-dependent pathways including recruitment of tumor-promoting macrophages [9], polarization of CD4+ T cells toward a pro-tumor phenotype [10], suppression of the IL-10 expression by CD8+ T cells [11] and NETosis promotion [12]. Furthermore, the contribution of complement system dysregulation to ocular disease pathogenesis is well described for age related macular degeneration [13–15], glaucoma [16, 17] and diabetic retinopathy [18, 19]. Ocular immune privilege is also maintained by tightly regulated, low-level complement activity [20].

A systematic analysis of 30 cancers revealed four prognostic patterns of complement gene expression: protective (pan-complement or C3-specific), tumor-promoting, or neutral. Notably, UM formed a distinct cluster, with complement expression strongly linked to poor prognosis [8]. This phenomenon has not been thoroughly investigated since. Therefore, in this study, we explored the role of complement components, complement regulators, and complement receptors (complement associated proteins, CAP) in UM hypothesizing that dysregulated complement signaling contributes to the immunosuppressive tumor microenvironment and promotes UM progression. Because UM research is often constrained by limited sample availability and the lack of suitable in vivo models, we employed a data integration strategy to gain insights with translational potential.

## Methods

For all analysis described we have investigated the role of CAP from the classical, lectin, and alternative pathways (C1QA, C1QB, C1QC, C1S, C1R, C2, C3, C4A, C4B, C5, C7, C8, C9, CFB, CFD, MASP1, MASP2) as well as regulatory components (CFI, CFH) and receptors (C3AR, C5AR1).

### TCGA data analysis

Publicly available transcriptomic and clinical data for pUM were obtained from TCGA via the cBioPortal for Cancer Genomics (https://www.cbioportal.org). Data were retrieved using the cBioPortal API and processed in R using cBioPortalData package. The uvm_tcga_pan_can-atlas_2018 dataset included mRNA expression (RNA-Seq V2 RSEM) values and clinical, molecular, and pathological annotations for UM patients. Expression values were log-transformed where appropriate for normalization and visualization. Patients were stratified based on chromosome 3 status (monosomy vs. disomy), progression and survival status.

### Primary UM and normal choroid tissue culture secretome analysis

Proteomic data from short-term primary cultures of pUM and normal choroid were obtained from a previously published study by Liverpool Ocular Oncology Research Group [21]. The dataset included label-free LC-MS/MS quantitative proteomics data of secreted proteins (secretome) derived from high-risk pUM (*n* = 10; defined by monosomy 3), low-risk pUM (*n* = 4; disomy 3), and normal choroid (*n* = 5). Levels of secreted CAP were extracted from the quantified secretome data. Protein identifiers were cross-referenced with known complement system components using UniProt annotations and manual curation. Protein abundance data were log10-transformed and normalized for inter-sample comparison. Only proteins identified with a false discovery rate (FDR) ≤ 1% were included in the analysis. Proteins were categorized into classically secreted, non-classical, or exosomal using SignalP, SecretomeP, and ExoCarta annotations. Differences in complement protein secretion: pUM vs. normal choroid, high-risk pUM vs. low-risk pUM were assessed.

### Single nucleus RNA- and single cell RNA-sequencing data analysis

Datasets were selected based on the following criteria: 1) publicly accessible from a trusted source with a peer-reviewed publication describing the original study’s design and methodology; 2) the data available in a format compatible with standard single-cell RNA‑sequencing (scRNA-seq) analysis pipelines, samples include annotations; 3) our analysis, did not reveal findings contradicting the original publication’s results or sample annotations. The scRNA-seq dataset of pUM and mUM was obtained from the Gene Expression Omnibus (GEO) (GSE139829) [22]. For the current analysis, we used three primary tumors (UMM069, UMM066, UMM065) and all three metastatic liver samples. snRNA-seq of the normal human eye posterior pole data were obtained from the Single Cell Portal (SCP2298) [23]. Only choroid and retina derived nuclei were included in downstream analyses. Normal liver scRNA-seq data from three healthy donors were obtained from GEO (GSE185477) [24]. Raw gene expression matrices and metadata were processed using the Seurat package (5.3.0) in R according to typical pipeline [25]. For the normal eye dataset, low-quality nuclei with fewer than 200 or higher than 6000 detected genes or mitochondrial gene content > 5% were removed. For the UM dataset, cells with fewer than 100 or more than 8000 detected genes or mitochondrial content > 10% were filtered out. For the normal liver dataset, cells with fewer than 100 detected genes or mitochondrial gene content > 50% were excluded. Gene expression values were normalized and scaled. Cell cycle phase variation was regressed out using CellCycleScoring function with the list of cell cycle markers available in Seurat package. Cell clusters were identified by examining the top 20 principal components across highly variable genes identified by the FindVariableFeatures function followed by Uniform Manifold Approximation and Projection (UMAP) for visualization in two dimensions. Louvain clustering algorithm was applied to group transcriptionally similar cells based on their shared nearest neighbors. Integration was performed to minimize batch effect. Cell cluster annotations for normal tissues datasets were taken directly from the metadata provided by the original authors. Marker gene signatures in UM datasets were scored using the UCell package (available from https://github.com/carmonalab/UCell). Cell type signatures were annotated as follows: Tumor cells: MLANA, MITF, DCT [22]; T-cells: CD3D, CD3E, CD8A [22]; B-cells: CD19, CD79A, MS4A [22]; Plasma cells: IGHG1, MZB1, SDC1, CD79A [22]; Monocytes and macrophages: CD68, CD163, CD14 [22]; Retinal pigment epithelium: RPE65 [22]; Photoreceptor cells: RCVRN [22]; Fibroblasts: FGF7, DCN, GREM1, PAMR1, COL1A1, COL3A1, COL6A1 [22, 26]; Endothelial cells: PECAM1, VWF [22]; Tregs: FOXP3, TNFRSF4, IKZF2, IL2RA [22]; Cytotoxic T-cells: PRF1, GZMA, GZMK, NKG7 [22]; Naïve T-cells: IL7R [22]; NK Cells: FGFBP2, CX3CR1 [22]; T effector memory cells: CD8A, ZNF683 [22]; myeloid-derived suppressor cells (MDSC): ITGAM, CD14, CD33 [27]. To compare overall CAP expression between pUM and normal eye and between pUM and mUM a pseudobulk approach was used. All plots were generated in R using Seurat and ggplot2.

### Intercellular communication analysis

To characterize intercellular communication CellChat v1 [28] was applied to infer global cell–cell signaling networks based on the expression of established ligand–receptor pairs. Subsequently, a more granular analysis was conducted using CellPhoneDB [29], implemented through the LIANA (LIgand–receptor ANalysis framework) platform [30]. 500 permutations were performed to generate null distributions.

### Pseudotime trajectory analysis

The investigation of the dynamic transcriptional changes and cell state transitions between normal liver fibroblasts and cancer-associated fibroblasts (CAF) from mUM were performed using pseudotime trajectory analysis with Monocle 3 package (https://github.com/cole-trapnell-lab/monocle3). Cells identified as fibroblasts were subsetted from mUM scRNA-seq dataset and fibroblasts and stellate cells were subsetted from normal liver scRNA-seq dataset. CAF subtypes were identified by markers defined by Cords L. et al. [31]. The resulting object was converted to a Monocle 3 CellDataSet for trajectory inference. Dimensionality reduction was performed using UMAP, and the trajectory graph was learned using Monocle’s reversed graph embedding algorithm. Cells were ordered in pseudotime to model gene expression dynamics along the inferred differentiation path.

### Immunohistochemical analysis

Formalin-fixed, paraffin-embedded (FPPE) human pUM tissue samples obtained from the Liverpool Ocular Oncology Biobank (HRA REC Ref 21/NW/0139) under project specific ethical approval (HRA REC Ref 11/NW/0568) were sectioned at 4 μm thickness. Sequential slides were prepared, with two adjacent sections per case: experimental and negative control slides. Immunohistochemistry (IHC) was performed using the Leica BOND-RXm fully automated staining system (Leica Biosystems, Germany) following manufacturer-recommended protocols. Experimental slides were incubated with anti-C5aR1 antibody (Abcam, catalog ab59390, dilution 1:250), negative control slides were incubated with rabbit IgG isotype control at matching concentration and conditions. Leica Bond Polymer Refine Detection System (DS9800) with 3,3’-Diaminobenzidine (DAB) were used to develop brown signal at the site of target antigen binding. Hematoxylin counterstain applied automatically for nuclear contrast. Digital slide scans were acquired using a VENTANA® DP 600 Digital Pathology Scanner (Roche Diagnostics, USA), slides were analyzed using QuPath software. Image analysis was carried out using the ‘Positive Cell Detection’ tool in QuPath. Detection thresholds were optimized on negative control slides to filter out low-level non-specific DAB staining (lower than 1+) and endogenous pigmentation (2+/3+). These settings were applied to C5aR1-stained slides, and true C5aR1-positive cell counts were estimated by subtracting 1+ counts in controls from those in experimental slides. Corrected counts were then used to assess associations with chromosome 3 status and tumor progression.

### Cell culture

The immortalized normal mouse colon fibroblast cell line mNDrmF3 was a kind gift from Prof. Buczacki (University of Oxford). Cells were maintained in Dulbecco’s Modified Eagle Medium (DMEM) supplemented with 10% fetal bovine serum (FBS) and 1% of penicillin and streptomycin (Pen Strep).

### Quantitative polymerase chain reaction (qPCR)

cDNA was synthesized using 1 µg of RNA and the Thermo Scientific™ Verso cDNA synthesis kit. C1S, C1R, C3 and RPS19 expression levels were quantified using qPCR (primer sequences represented in Supplement table 1). Three technical replicates were plated. All the qPCR plates were run on the StepOne Real-Time PCR system. Expression was normalized to the Beta-actin gene and calculated as 2^-ΔCt.

### Statistical analysis

Group comparisons were performed using the Mann–Whitney U test, with Bonferroni adjustment for multiple comparisons. The *p*-values < 0.05 were considered significant. Survival analysis used the Kaplan-Meier method, with groups defined by expression levels relative to one standard deviation above the mean. Significance was assessed by log-rank test, and hazard ratios estimated via Cox regression. Analyses were conducted in R (v4.5.0) using survival, survminer, and ggplot2 packages.

## Results

### In pUM, complement gene expression is biased toward early pathway components over terminal membrane attack complex elements

**Fig. 1:**
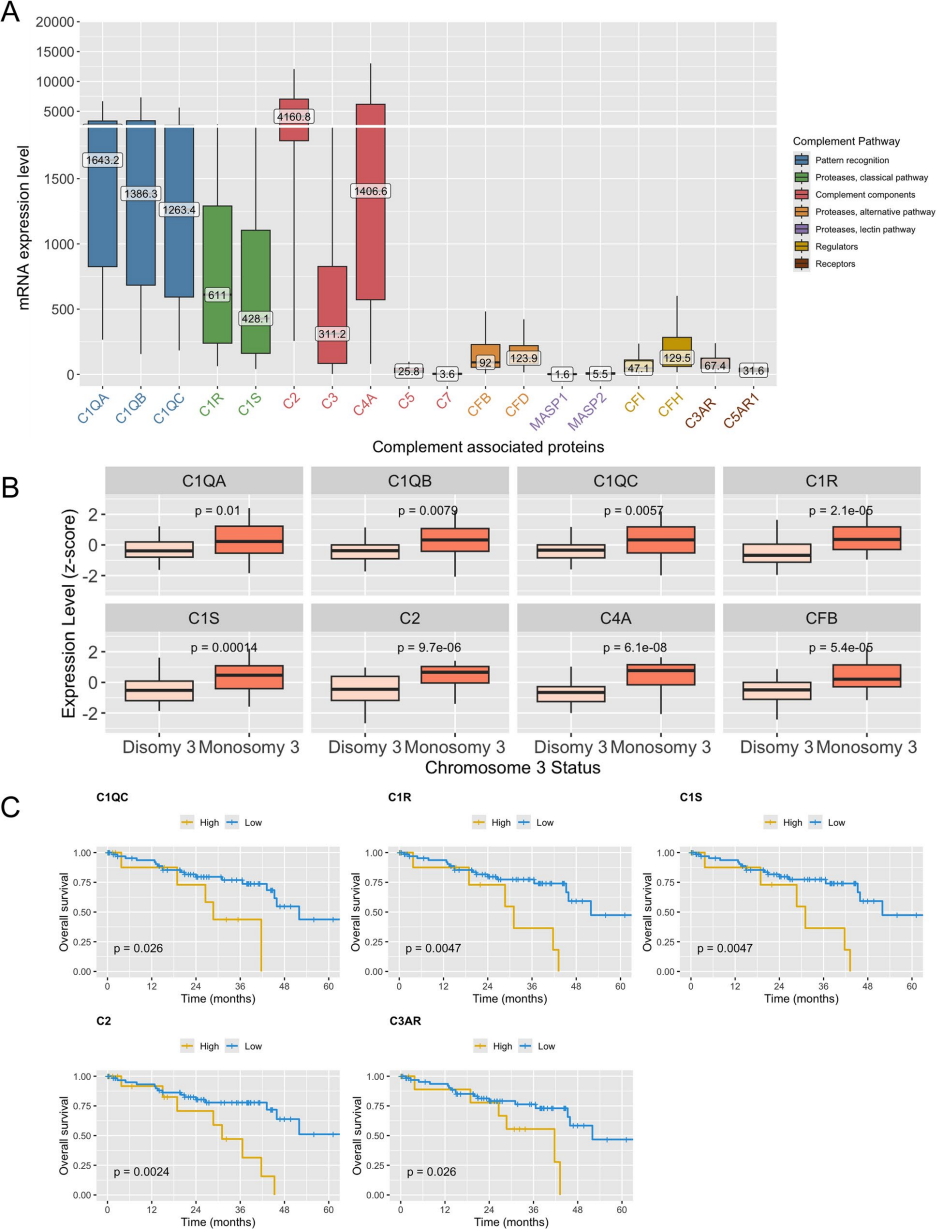
RNA expression analysis of complement associated proteins in primary uveal melanoma (pUM) TCGA data. **A** – batch-normalized mRNA expression of complement associated proteins in the TCGA pUM cohort. Boxes represent the interquartile range, whiskers indicate the range of values excluding outliers, and median expression levels are labeled at the median line. **B** – mRNA expression levels of complement associated proteins (z-scores relative to all samples) in low-risk (disomy 3) and high-risk (monosomy 3) groups. Boxes represent the interquartile range, whiskers indicate the range of values excluding outliers, and median expression levels are labeled at the median line. Mann–Whitney U test compared risk groups. **C** – overall survival in TCGA cohort depending on complement associated protein expression levels. High expression (high) – ≥ 1 standard deviation above mean, low expression (low) – < 1 standard deviation above mean. Log-rank test compared survival in groups. Only statistically significant results are shown

The mRNA transcripts of all evaluated CAP were detected in 100% of pUM samples obtained from the TCGA Rare Tumor Project, except for C8, C9, and C4B, which were undetectable (Fig. 1A). Gene expression patterns indicated a predominant involvement of the classical complement pathway, followed by the alternative pathway, while components of the lectin pathway were expressed at lower levels (Fig. 1A). The highest expression was observed for C2, C1QA, C4A and C1QC. In contrast, C5 and C7 (terminal complement components), as well as CFB and CFD (alternative pathway proteases), MASP1/MASP2 (lectin pathway proteases), and complement regulators (CFI, CFH) were expressed at relatively low levels. Notably, C3AR expression was approximately twice that of C5AR1. Next, we examined CAP expression in relation to chromosome 3p copy-number status since loss of chromosome 3p is typically associated with high-risk tumors. Significant upregulation in the high-risk group was observed for C1QA, C1QB, C1QC, C1S, C1R, C2, C4A, and CFB (Fig. 1B). Survival analysis revealed that lower expression of several CAP, including C1QC, C1R, C1S, C2, and C3AR, was significantly associated with longer OS (Fig. 1C).

### C1R and C1S are highly secreted by high-risk tumors

Since complement components are typically secreted proteins, it is important to evaluate not only their cellular expression but also their abundance in the secreted milieu. Accordingly, we investigated the levels of complement components within the proteome secreted by pUM and normal choroid (NC). In the secretome derived from high- and low-risk pUM and NC, a total of 14 complement components were detected (Table 1).

**Table 1:** Complement components identified in the secretome of pUM and NC

Protein	Highest in	Lowest in	Peptide count	Unique peptides
C1QA	Low risk	Normal*	1	1
C1QC	Low risk	Normal	2	2
C1S	High risk	Normal*	10	10
C1R	High risk	Normal*	6	5
C2	Low risk	Normal*	3	3
C3	High risk	Normal*	7	6
C4A	High risk	Normal*	10	10
C5	Low risk	Normal*	1	1
C7	High risk	Normal*	2	2
C8	High risk	Normal*	1	1
C9	High risk	Normal*	1	1
CFB	High risk	Normal*	1	1
CFI	Low risk	Normal*	5	5
CFH	High risk	Normal	10	10

*p < 0.05

**Fig. 2:**
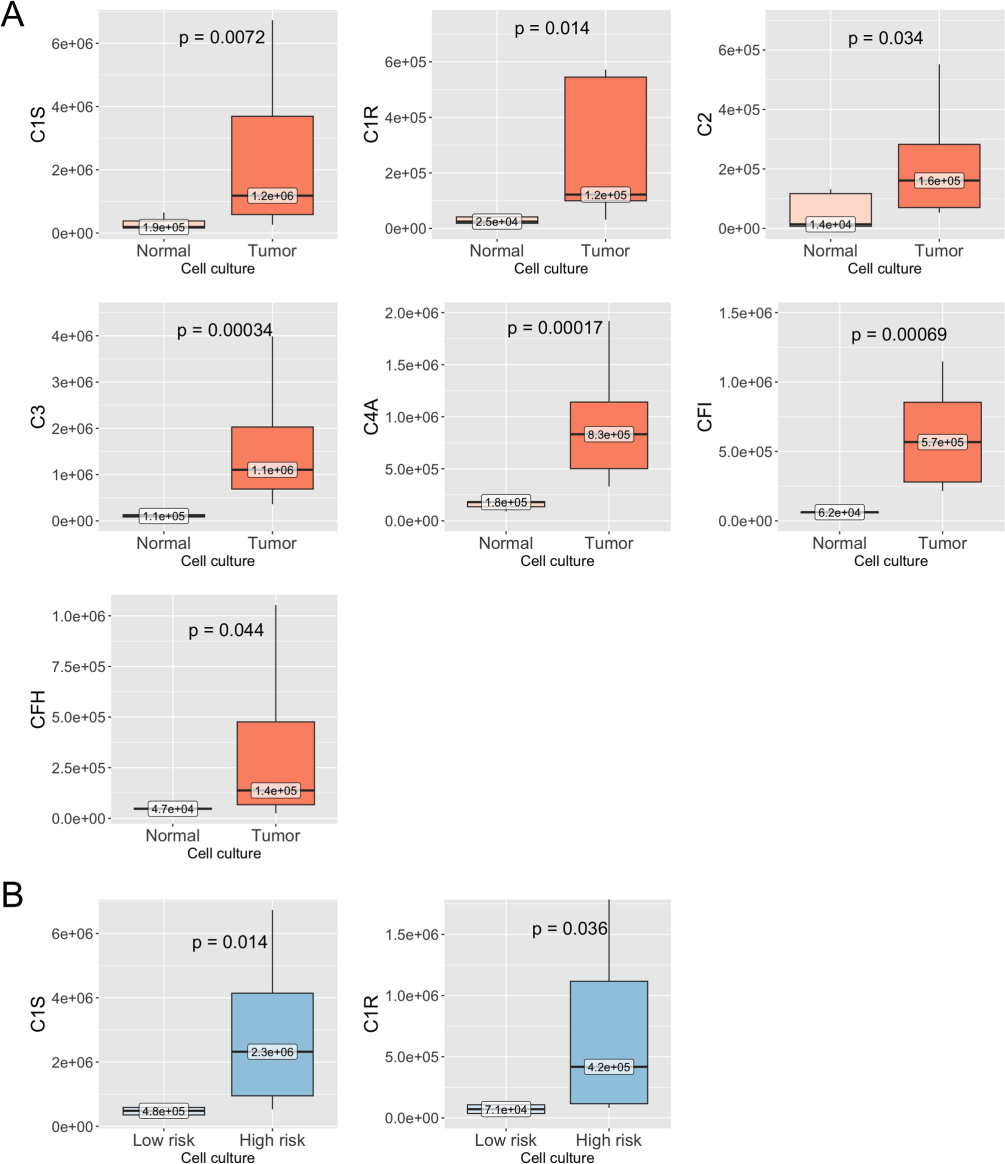
Levels of complement associated proteins in primary uveal melanoma (pUM) and normal choroid tissue cultures secretome. **A**– complement associated protein levels in pUM (tumor) compared to normal choroid (normal) cell culture media shown as boxplots. Groups compared by Mann–Whitney U test. Boxes represent the interquartile range, whiskers indicate the range of values, and median levels are labeled at the median line. Statistically significant differences are shown. **B**– complement associated protein levels in the low- and high-risk pUM cell culture media shown as boxplots. Boxes represent the interquartile range, whiskers indicate the range of values, and median levels are labeled at the median line. Groups compared by Mann–Whitney U test. Statistically significant differences are shown

For downstream analysis, we only included proteins that were detected by three or more unique peptides. The secretory profiles were comparable between tumor and normal samples, with C1S, C4A, and C3 exhibiting the highest abundance (Supplement Figure 1A, 1B). Levels of all detected complement proteins were significantly higher in tumor samples compared to NC (Fig. 2A). Furthermore, C1S and C1R were significantly elevated in high-risk tumors compared to low-risk samples (Fig. 2B). Since C1Q components were not detected in secretome data in either tumor or NC samples, we hypothesized that this absence could be due to macrophage depletion caused by the cell culture methodology. Indeed, no macrophage-associated proteins (IDO1, LSP1, FCN1, CCL4, CCL3, CXCL3) [32] were detected in the secretomes. Additionally, fibroblast markers such as COL18A1, COL4A2, COL6A1, COL4A1, COL1A1, COL1A2, COL3A1, COL6A3, COL5A2, FSTL1, VIM, and FN1 [33], detected by three or more peptides, were found in the pUM secretome, indicating the possible contribution of fibroblasts to the secretory profile of the culture. We have further validated C1S, C1R, and C3 expression in normal fibroblasts in vitro (Supplement Figure 2A).

### Fibroblasts and macrophages as predominant sources of CAP in primary and metastatic uveal melanoma

**Fig. 3:**
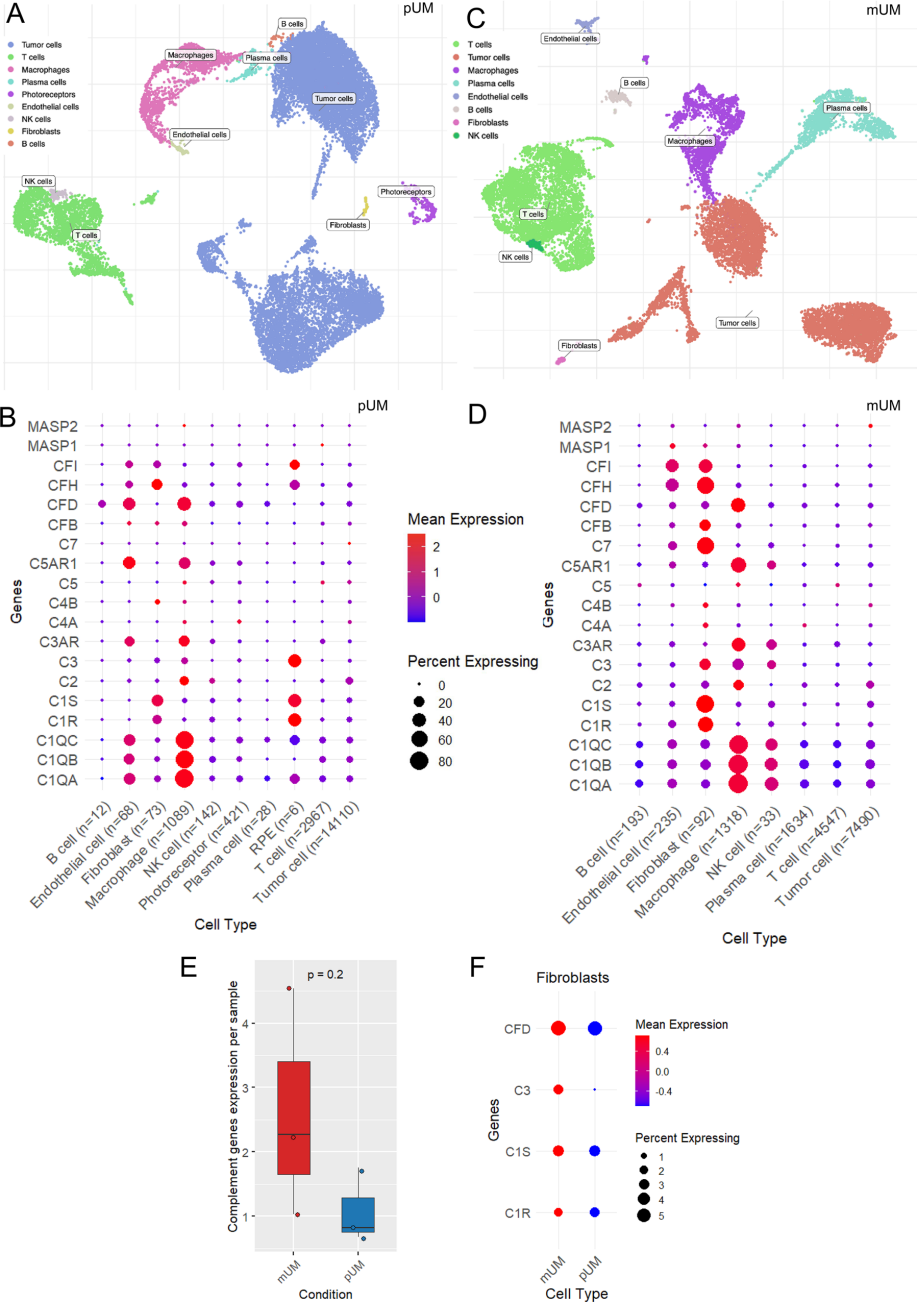
Complement expression in primary and metastatic UM by scRNA-seq. **A** – UMAP plot of 18,916 pUM cells distributed by annotated unsupervised clustering in pUM cases. **B** – dot plot showing the expression of complement associated proteins across cell types in pUM. Mean expression reflects relative expression compared to other cell types and genes represented on the graph. Expression levels are log-normalized and scaled. The percent expressing indicates how widespread the expression is within each cell type. **C** – UMAP plot of 15,544 mUM cells distributed by annotated unsupervised clustering in mUM cases. **D** – dot plot showing the expression of CAP across cell types in mUM. Mean expression reflects relative expression compared to other cell types and genes represented on the graph. Expression levels are log-normalized and scaled. The percent expressing indicates how widespread the expression is within each cell type. **E** – boxplot displaying the mean expression levels of CAP in mUM and pUM, aggregated per sample using a pseudobulk approach. Each dot represents an individual sample: 3 samples in the mUM cohort and 3 samples in the pUM cohort. Groups compared by Mann–Whitney U test. **F** – dot plot illustrating the difference in expression of CAP genes by fibroblasts from mUM and pUM. Mean expression reflects relative expression compared to other cell types and genes. Expression levels are log-normalized and scaled. The percent expressing indicates how widespread the expression is within each cell type. CAP – complement associated proteins, RPE – retinal pigmented epithelium, MDSC – myeloid derived suppressor cells, pUM – primary uveal melanoma, mUM – metastatic uveal melanoma

To further investigate the cellular sources of CAP at a higher resolution, we analyzed publicly available UM scRNA-seq data. Following quality control and filtering, the pUM dataset comprised 18,916 cells and 35,694 features across three samples (Fig. 3A). Despite the predominance of tumor cells, the primary producers of CAP were endothelial cells, fibroblasts and macrophages (Fig. 3B). Amongst the CAP significantly associated with prognosis in bulk TCGA data and secretome analyses (Fig. 1C), members of the C1Q complex were highly expressed in endothelial cells and macrophages. C1R and C1S were mainly expressed in fibroblasts, while C2 expression was enriched in macrophages (Fig. 3B). Compared to snRNA-seq data from the normal eye posterior pole, the overall expression of CAP was lower in pUM with the pseudobulk approach (Supplement Figure 3A). This is in contrasts to the secretome data where tumor samples secreted higher levels of CAP than NC. In the normal eye, fibroblasts and macrophages were the main sources of CAP expression, with high C3 and CFH levels possibly supporting ocular immune privilege (Supplement Figure 3B).

The mUM scRNA-seq dataset comprised 15,544 cells and 33,694 features across three samples following quality control and filtering. Tumor cells represented the dominant population, while fibroblasts were present in smaller proportions (Fig. 3C). Fibroblasts and macrophages were identified as the primary expressors of CAP in mUM (Fig. 3D). The expression pattern was consistent with previous observations: members of the C1Q complex were highly expressed in macrophages, while C1R and C1S were predominantly expressed in fibroblasts. C3 expression was observed in both fibroblasts and macrophages in contrast with pUM where C3 was not prominent (Fig. 3D).

CAP expression levels were overall higher in mUM compared to pUM (Fig. 3E), with particularly elevated expression of C1R, C1S, C3, and CFD in fibroblasts consistent with the secretome data (Fig. 3F). Given previous findings indicating that complement components contribute to the formation of metastatic niches via MDSC recruitment [34], and that C3-primed fibroblasts may shift to a proinflammatory phenotype [35], we hypothesize that similar mechanisms may be involved in UM metastatic progression.

### Fibroblasts can support an immunosuppressive tumor microenvironment through complement associated signaling

**Fig. 4:**
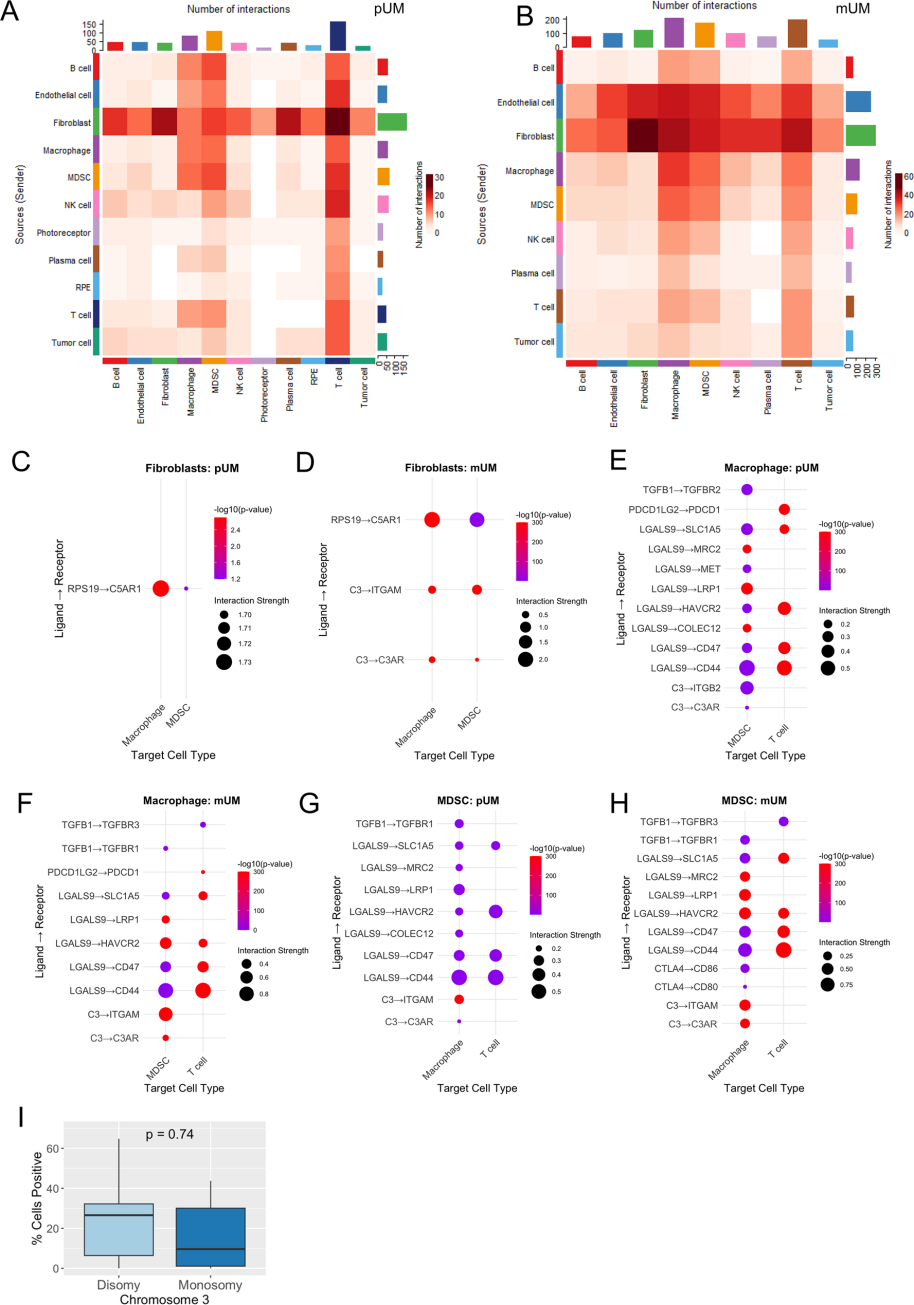
Interaction analysis in primary and metastatic UM. **A** – Heatmap illustrating cell–cell interaction analysis with CellChat in pUM samples. Fibroblasts emerge as the predominant source of outgoing signals, while macrophages, MDSC, and T cells display the highest number of interactions. **B** – Heatmap illustrating cell–cell interaction analysis with CellChat in mUM. **C** – complement associated interactions derived from fibroblasts withing pUM microenvironment. Dot plot represents ligand-receptor interactions involving CAP where ligands are derived from fibroblasts. Significant interactions (*p* < 0.05) determined by CellPhoneDB permutation test (500 iterations) are shown. Dot size represents how strongly the pair is expressed relative to the other interactions. **D** – complement associated interactions derived from fibroblasts withing mUM microenvironment. Significant interactions (*p* < 0.05) determined by CellPhoneDB permutation test (500 iterations) are shown. Dot size represents how strongly the pair is expressed relative to the other interactions. **E** – complement associated and immunosuppression promoting interactions derived from macrophages in pUM. Significant interactions (*p* < 0.05) determined by CellPhoneDB permutation test (500 iterations) are shown. **F** – complement associated and immunosuppression promoting interactions derived from macrophages in mUM. Significant interactions (*p* < 0.05) determined by CellPhoneDB permutation test (500 iterations) are shown. **G** – complement associated and immunosuppression promoting interactions derived from MDSC in pUM. Significant interactions (*p* < 0.05) determined by CellPhoneDB permutation test (500 iterations) are shown. **H** – complement associated and immunosuppression promoting interactions derived by MDSC in mUM. Significant interactions (*p* < 0.05) determined by CellPhoneDB permutation test (500 iterations) are shown. **I** – IHC analysis of C5AR1 expression in 15 pUM human samples stratified by risk of progression (low- and high-risk tumors defined by chromosome 3 status). Boxes represent the interquartile range, whiskers indicate the range of values excluding outliers, and median levels are labeled at the median line. Mann–Whitney U test compared risk groups. pUM – primary uveal melanoma, mUM – metastatic uveal melanoma, CAP – complement associated proteins, RPE – retinal pigmented epithelium, MDSC – myeloid derived suppressor cells, RPS19 – ribosomal protein S19

Cell–cell interaction analysis of the pUM and mUM datasets revealed that fibroblasts were the predominant source of outgoing signals across all intercellular interactions, not limited to those involving CAP. In contrast, macrophages, T cells, and MDSC were identified as the main recipients, exhibiting the highest number of interactions. (Fig. 4A, B). CAF have been shown to modulate the tumor immune microenvironment in both mouse models and human cancers [36–38] and contribute to pre-metastatic niche formation [39, 40]. Fibroblasts are also known to regulate inflammation via complement production [35, 41]. Complement components C3a and C5a have been implicated in the recruitment and activation of MDSC in various cancer models, including breast [34], lung [42] and cervical cancers, however no reports of their involvement in UM are known [43]. Although C5 expression was nearly absent in pUM, CellPhoneDB analysis of complement-related interactions revealed a notable fibroblast–MDSC and fibroblast–macrophage interaction involving the ribosomal protein RPS19 and the receptor C5AR1 (Fig. 4C, D). The IHC analysis of 15 human samples of pUM confirmed pUM C5AR1 positivity (Supplement figure 4) but revealed no statistically significant difference of C5AR1 expression between low- and high-risk patients defined by chromosome 3 status (Fig. 4I). The RPS19–C5AR1 interaction was as prominent in mUM, as it was in pUM (Fig. 4C, D), consistent with the lack of difference in C5aR1 expression between low- and high-risk pUM in human tumor samples on IHC. RPS19 was shown to be secreted in high- and low-risk pUM primary tissue culture (Supplement figure 5) and expressed by fibroblasts in vitro (Supplement figure 2B).

RPS19–C5AR1 interaction has been previously suggested to induce immunosuppressive TGF-β signaling [44]. We therefore hypothesized that it could similarly lead to TGF‑β signaling in the UM microenvironment. Indeed, both MDSC and macrophages were found to express TGFB1, potentially contributing to the immunosuppressive microenvironment in pUM (Fig. 4E, G) and mUM (Fig. 4F, H), although this remains to be formally proven. Additionally, T cell subtypes within the primary and metastatic tumors exhibited features of immune suppression, as indicated by the expression of immune checkpoints (Supplement Figure 6). MDSC and macrophages were predicted to interact with T cells through PD-1, TIM-3 (HAVCR2), and LGALS9, engaging with CD44, CD47, and SLC1A5 (Fig. 4E-H) known to inhibit immune responses [45–47]. Additionally, MDSC were predicted to interact with macrophages through C3 – ITGAM interactions (Fig. 4G, H). C3 associated signaling was more prominent in the metastatic tumors with fibroblast–MDSC and fibroblast–macrophage interactions enriched through C3–C3AR and C3—ITGAM signaling (Fig. 4D).

**Fig. 5:**
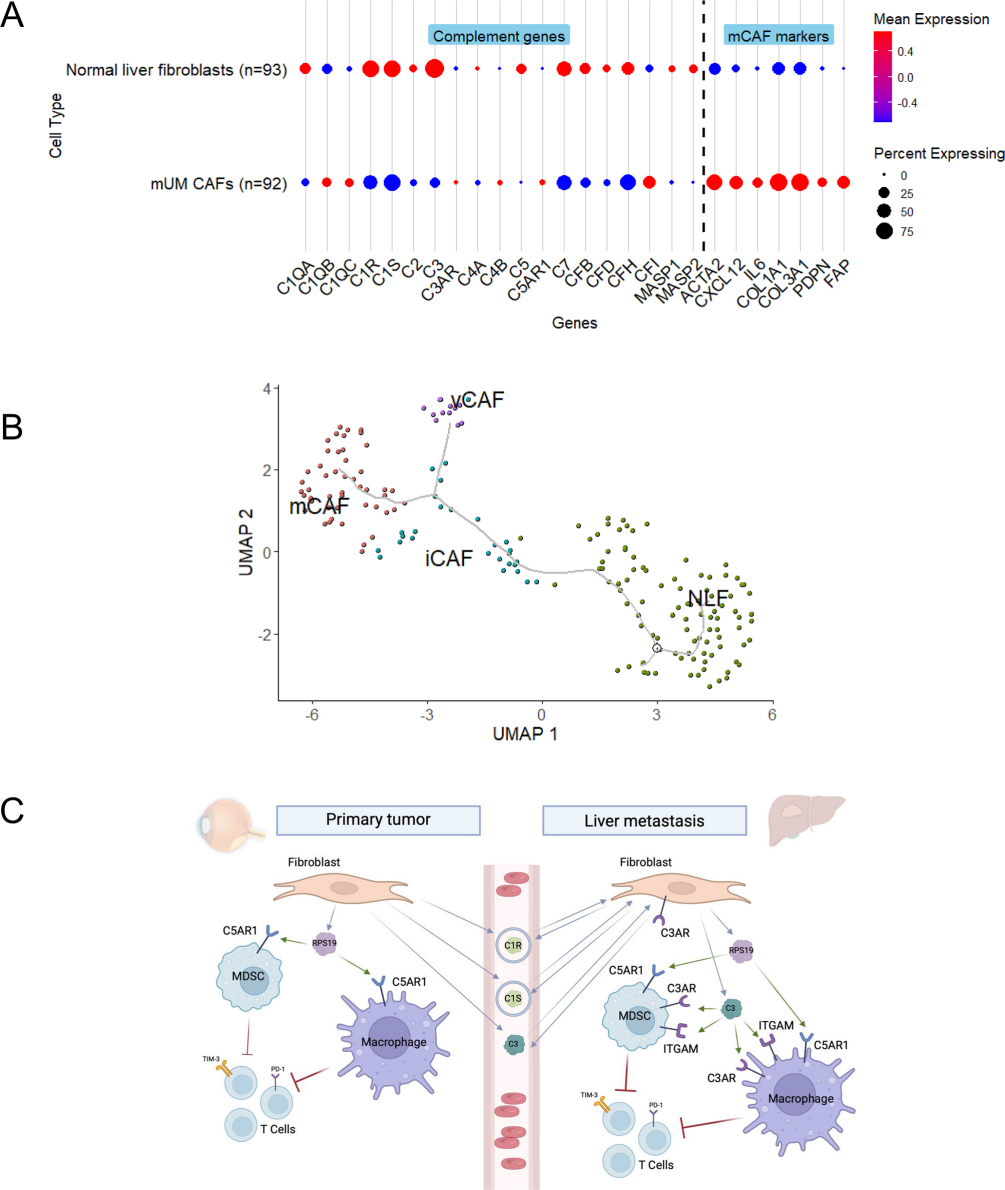
Analysis of cancer associated fibroblasts in metastatic UM. **A** – dot plot illustrates the differences in expression of complement associated proteins and mCAF markers between normal liver fibroblasts and mUM cafs. Mean expression reflects relative expression compared to other cell types. Expression levels are log-normalized and scaled. The percent expressing indicates how widespread the expression is within each cell type. **B** – trajectory analysis of normal liver fibroblasts (NLF), and mUM CAF subtypes. UMAP plot showing clusters of NLF and mUM CAF distributed along pseudotime, with NLF set as the starting point. The trajectory illustrates a phenotypic transition from NLF to iCAF, followed by divergence into either mCAF or vCAF states. **C** – working model derived from key findings from scRNA-seq and secretome analyses of complement associated protein expression and secretion in pUM and mUM. Fibroblasts may orchestrate the establishment of an immunosuppressive microenvironment through C3–C3AR, C3–ITGAM, and RPS19–C5AR1 signaling, which promotes MDSC recruitment and differentiation, as well as macrophage M2-like polarization. In turn, these changes inhibit T cell function via immune checkpoint pathways. C1R and C1S are secreted via exosomes and, along with C3, may contribute to the education of liver fibroblasts and the formation of a pre-metastatic niche. iCAF – inflammatory cancer associated fibroblasts, mCAF – matrix cancer associated fibroblasts, vCAF – vascular cancer associated fibroblasts, NLF – normal liver fibroblasts, RPS19 – ribosomal protein S19, MDSC – myeloid derived suppressor cells

To further investigate the role of fibroblasts, we compared the expression of CAP between mUM-derived fibroblasts and normal hepatic fibroblasts, including stellate cells, both of which can transition into CAFs [48, 49]. Unexpectedly, most CAP were significantly upregulated in normal liver fibroblasts compared to mUM, with significant differences in C1QA, C1R, C1S and C3 (*p* < 0.001) expression. mUM CAFs showed significantly increased expression of genes involved in extracellular matrix deposition and remodeling, such as COL1A1, COL1A2, ACTA2, and FAP (*p* < 0.001), indicating a phenotypic shift from immune-active fibroblasts to matrix-producing CAF in established metastases (Fig. 5A). Trajectory analysis further showed the iCAF phenotype to be a transitional state from normal liver fibroblasts to mCAF (Fig. 5B).

## Discussion

This study comprehensively characterized the complement system in pUM and mUM using integrated proteomic, transcriptomic, and single-cell approaches. Although UM is a rare tumor and research is often limited by sample availability, we demonstrate that valuable insights can be gained from publicly available datasets. Our findings revealed significant changes in complement-related genes across UM compartments, implicating complement activation in tumor progression and immune suppression.

Analysis of CAP gene expression in pUM TCGA data revealed a predominant bias towards increased expression of genes in the classical pathway and their upregulation in high-risk tumors, indicating an association between classical complement gene expression and more aggressive disease. Terminal pathway components showed low or no expression, suggesting complement activation in pUM may favor initiation over full cascade progression, indicating localized immune modulation rather than strong lytic activity. Survival analysis confirmed the prognostic significance of complement gene expression with high levels of early classical pathway genes (C1QC, C1R, C1S, C2) and C3AR being associated with worse overall survival.

At a bulk tissue level, CAP appeared downregulated in pUM compared to the normal eye posterior pole. However, primary tumor cultures secreted higher CAP levels than the normal choroid. A similar discrepancy has been observed in lung cancer [50]. This may reflect post-transcriptional regulation, where tumor cells enhance translation or secretion efficiency despite lower transcript levels, highlighting the importance of integrating transcriptomic and proteomic data in complement studies. Although the underlying mechanisms remain unclear, this mismatch could be leveraged for clinical cancer monitoring, as high serum complement levels may indicate progression risk, treatment resistance or minimal residual disease.

Complement gene expression was elevated in mUM compared to pUM, supporting the hypothesis that complement upregulation contributes to metastatic niche establishment or maintenance. Notably, C1S and C1R were secreted at significantly higher levels in high-risk pUM compared to low-risk tumors, pointing to a potential role in disease progression. Their presence in exosomes [21], further supports their potential involvement in pre-metastatic niche formation. These findings also reinforce prior reports that complement activation contributes to the creation of a permissive metastatic microenvironment in breast cancer lung metastases [51].

At the cellular level, fibroblasts and macrophages, rather than tumor cells, were the primary source of CAP in pUM and mUM. Fibroblasts were also identified as the dominant source of signaling in both tumor stages, while macrophages, MDSC and T cells were the cell types with most numerous predicted interactions. UM has traditionally been classified as an immune-cold tumor. However, scRNA-seq data reveal the presence of intratumoral T cells, including cytotoxic subsets expressing multiple immune checkpoints, such as TIGIT, PD-1, LAG-3, TIM-3 and CTLA-4, indicating a functionally exhausted phenotype. Ligand–receptor analysis of complement-related interactions further revealed a fibroblast–MDSC–macrophage signaling network that may contribute to the immunosuppressive environment establishment through RPS19–C5AR1 axis and T cell activity modulation through PD-1 and TIM-3 pathways, as well as LGALS9-mediated signaling [45–47] (Fig. 5C). Our data suggest that signaling networks in mUM are more complex than in pUM, with C3-related pathways having a more prominent role. Fibroblast interactions with MDSC and macrophages through C3–ITGAM and C3–C3AR pathways may contribute to MDSC recruitment [52, 53], differentiation [54], and macrophages skewing towards M2-like polarization [55, 56]. This immunosuppressive signaling may be further amplified by MDSC–macrophage interactions via the same C3–ITGAM and C3–C3AR axes (Fig. 5C). These findings are consistent with those for gastric cancer peritoneal metastases where immunosuppressive microenvironment establishment was also orchestrated by mCAF through C3–C3AR signaling [57]. Given that inhibition of the C3–C3AR axis enhanced the efficacy of immune checkpoint blockade in vivo for gastric cancer peritoneal metastases [57] this approach could be also beneficial in UM.

Trajectory analysis indicated the transition from normal liver fibroblasts to mCAF through iCAF. Interestingly, a shift from immune-active CAFs to matrix-producing CAFs was detected in established UM metastases. It is tempting to speculate that CAF could be educated by complement signaling at the early stages of metastasis establishment and could be therapeutically reprogrammed to a normal state at the iCAF stage. We also identified the vCAF subpopulation, which points to a hypoxic microenvironment, warranting further investigation into its role in the intratumoral immune suppression.

To our knowledge, this is the first study to systematically explore the role of the complement system in UM. Our findings demonstrate that UM is better characterized by an immunosuppressive microenvironment rather than an immune-cold one and CAP may contribute to the establishment and maintenance of this immunosuppressive state. The identification of druggable complement targets such as C1S and C3 paves the way for therapeutic innovation. At present, reliable animal models to assess the efficacy of complement-targeted therapies in preventing UM metastasis or treating mUM are lacking. Nevertheless, these drugs are already well-established and widely used in both ophthalmological and systemic indications [58–60]. Their repurposing for high-risk or metastatic UM could offer a promising route toward more effective treatments. A Phase II clinical trial of C3 targeting agents could be considered, offering a promising strategy for addressing the unmet need in metastatic UM treatment.

## Electronic supplementary material

Below is the link to the electronic supplementary material.

Supplementary material 1

## Data Availability

snRNA-seq of normal eye data are available from the Single Cell Portal (SCP2298). scRNA-seq of primary and metastatic uveal melanoma data are available from the Gene Expression Omnibus (GSE139829). Normal liver scRNA-seq data are available from Gene Expression Omnibus (GSE185477). Secretome data are available from the Liverpool Ocular Oncology Research Group (HK, SEC) on reasonable request.
