# Supplementary material for: The complement system contributes to the immunosuppressive microenvironment of uveal melanoma
Source: J Transl Med. 2026 Mar 9;24:612. doi: 10.1186/s12967-026-07910-y (PMC13130498; doi:10.1186/s12967-026-07910-y)
Supplement: Supplementary file 1 — Supplementary material 1 [file 12967_2026_7910_MOESM1_ESM.docx]

***Supplement Table 1 – Primer sequences for qPCR***

| Gene symbol  (Mus musculus) |  | Sequence (5’-3’) | Length (bp) |
| --- | --- | --- | --- |
| C1S | Forward | ACACTGAGCCCTGGGTGGT | 19 |
|  | Reverse | AACAGGACCAAACACCACATCCC | 23 |
| C1R | Forward | CCCGGTGTCTGCCAGTGTG | 19 |
|  | Reverse | CTCGCCCGTGGGTAGTGGT | 19 |
| C3 | Forward | GAGCGAAGAGACCATCGTACT | 21 |
|  | Reverse | TCTTTAGGAAGTCTTGCACAGTG | 23 |
| RPS19 | Forward | GCCCGGAGTTACTGTAAAAGACGTT | 25 |
|  | Reverse | ACTGTGTCCACCCATTCGGG | 20 |
| Beta-actin | Forward | CCACTGTCGAGTCGCGTCCA | 20 |
|  | Reverse | TCCAGGCATCGAAAAGCCCGA | 21 |
| 18S | Forward | CGGAAAATAGCCTTCGCCATCAC | 23 |
|  | Reverse | ATCACTCGCTCCACCTCATCCT | 22 |


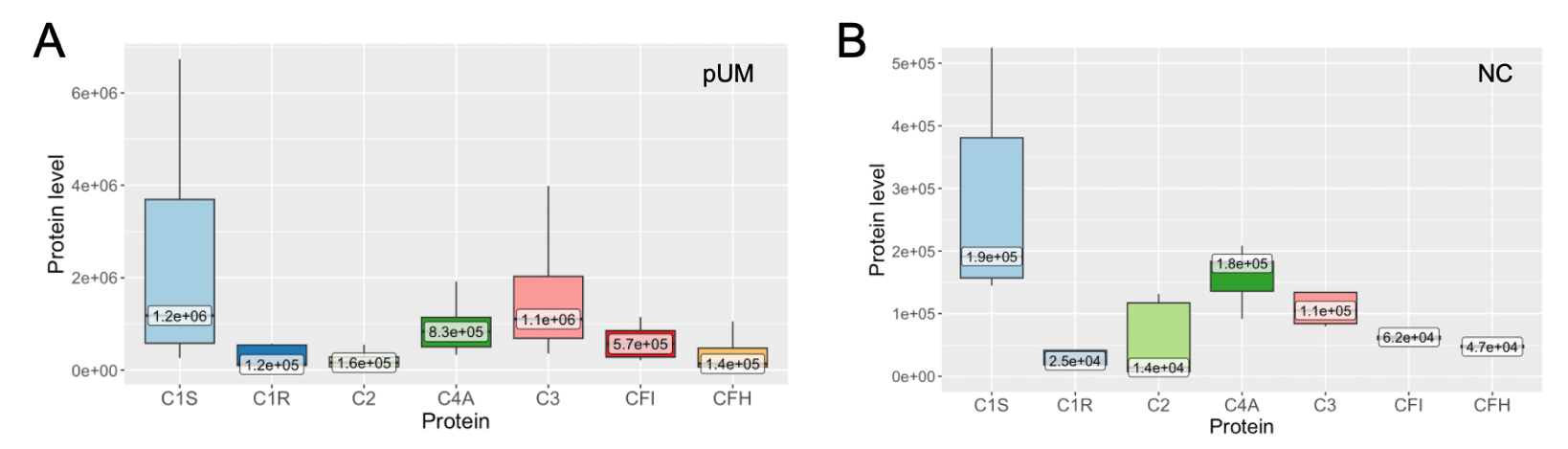


***Supplement Figure 1 – Complement associated protein levels in primary uveal melanoma (pUM) and normal choroid (NC) tissue cultures secretomes.***

**A** – Levels of complement associated proteins in the pUM cell culture media are shown as boxplots. **B** –Levels of complement associated proteins in the NC cell culture media shown as boxplots. Boxes represent the interquartile range, whiskers indicate the range of values, and median levels are labeled at the median line.


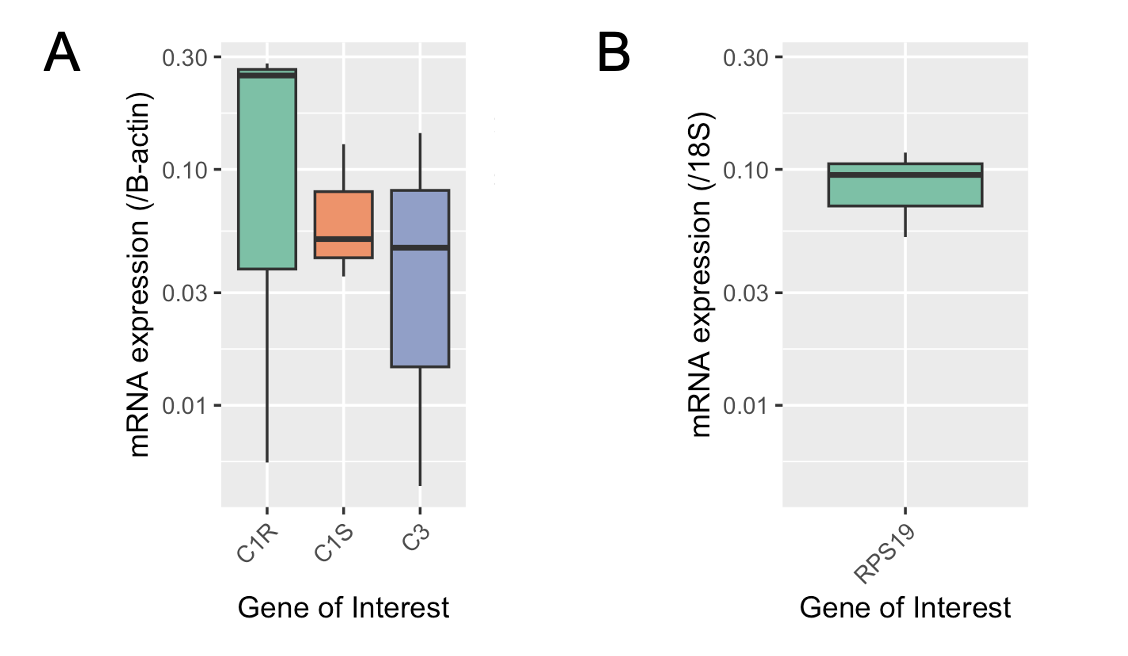


***Supplement Figure 2 – C1R, C1S, C3, RPS19 expression in fibroblasts detected by qPCR.***

**A –** C1R, C1S, and C3 mRNA expression was assessed in normal mouse fibroblasts (mNDrmF3 cell line) by qPCR in three biological replicates. Expression of each gene was normalized to the Beta-actin gene, calculated as 2^-ΔCt. **B** – RPS19 mRNA expression was assessed in normal mouse fibroblasts (mNDrmF3 cell line) by qPCR in three biological replicates. Expression was normalized to 18S gene, calculated as 2^-ΔCt. In the boxplots, boxes represent the interquartile range, whiskers indicate the range of values, and median levels are labeled at the median line.


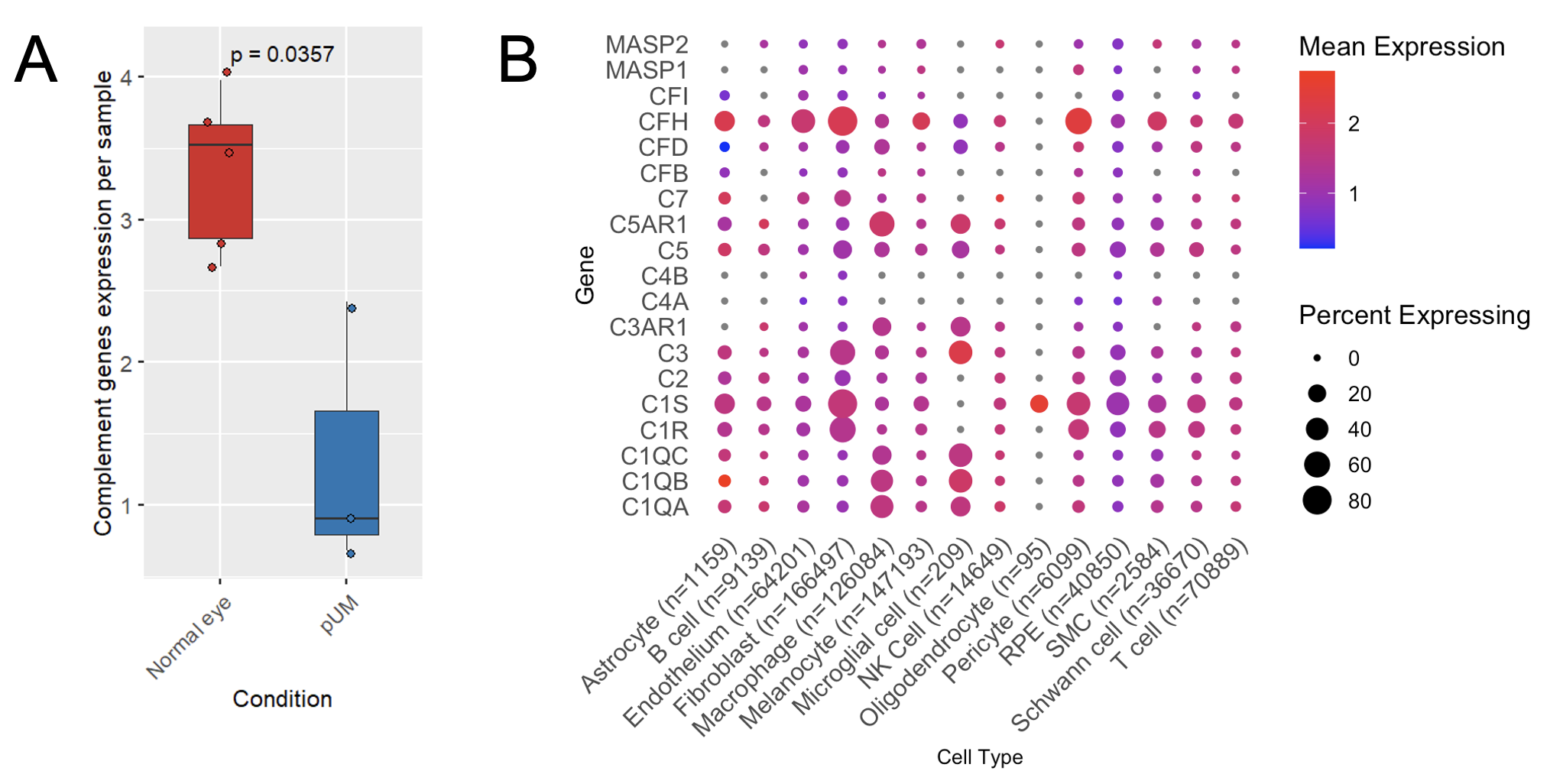


***Supplement Figure 3 – Single-nucleus RNA sequencing of the normal eye posterior pole data analysis.***

**A** – Box plot displaying the mean expression levels of complement associated proteins in the choroid of the normal eye posterior pole and in pUM, aggregated per sample using a pseudobulk approach. Each dot represents an individual sample: 4 samples in the normal eye cohort and 3 samples in the pUM cohort. Groups compared by Mann–Whitney U test. **B** – Dot plot illustrating the expression of complement associated proteins across different cell types in the normal eye posterior pole. Mean expression reflects relative expression compared to other cell types. Expression levels are log-normalized and scaled. The percent expressing indicates how widespread the expression is within each cell type.

***
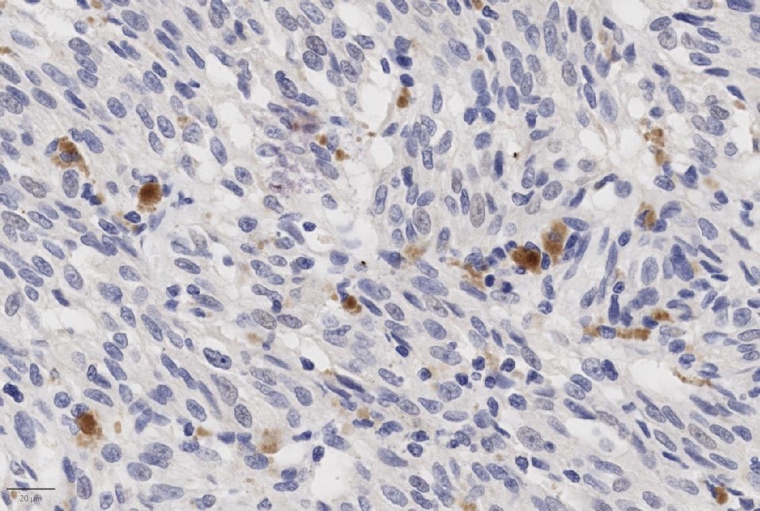

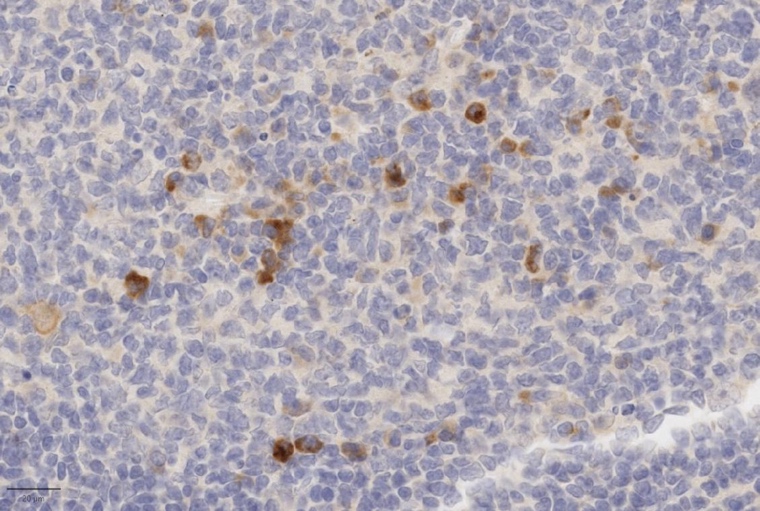
***

B

A

***Supplement Figure 4 – C5AR1 immunohistochemistry in primary uveal melanoma.***

Representative slides of immunohistochemical staining for C5AR1 in primary uveal melanoma (pUM). **A** – Positive control using tonsil tissue. **B** – pUM tissue section demonstrating positive C5AR1 immunoreactivity. Staining was performed using a DAB chromogen (brown) with a hematoxylin counterstain (blue).


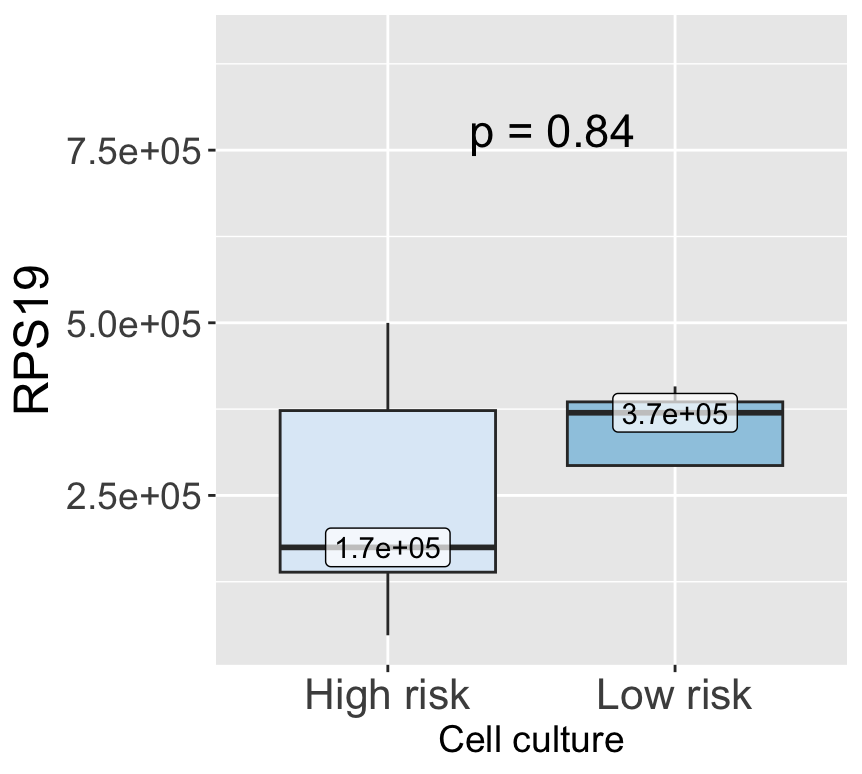


***Supplement Figure 5 – RPS19 in primary UM tissue culture secretome.***

RPS19 levels in the low- and high-risk pUM cell cultures media shown as boxplots. Boxes represent the interquartile range, whiskers indicate the range of values, and median levels are labeled at the median line. Groups compared by Mann–Whitney U test.

***
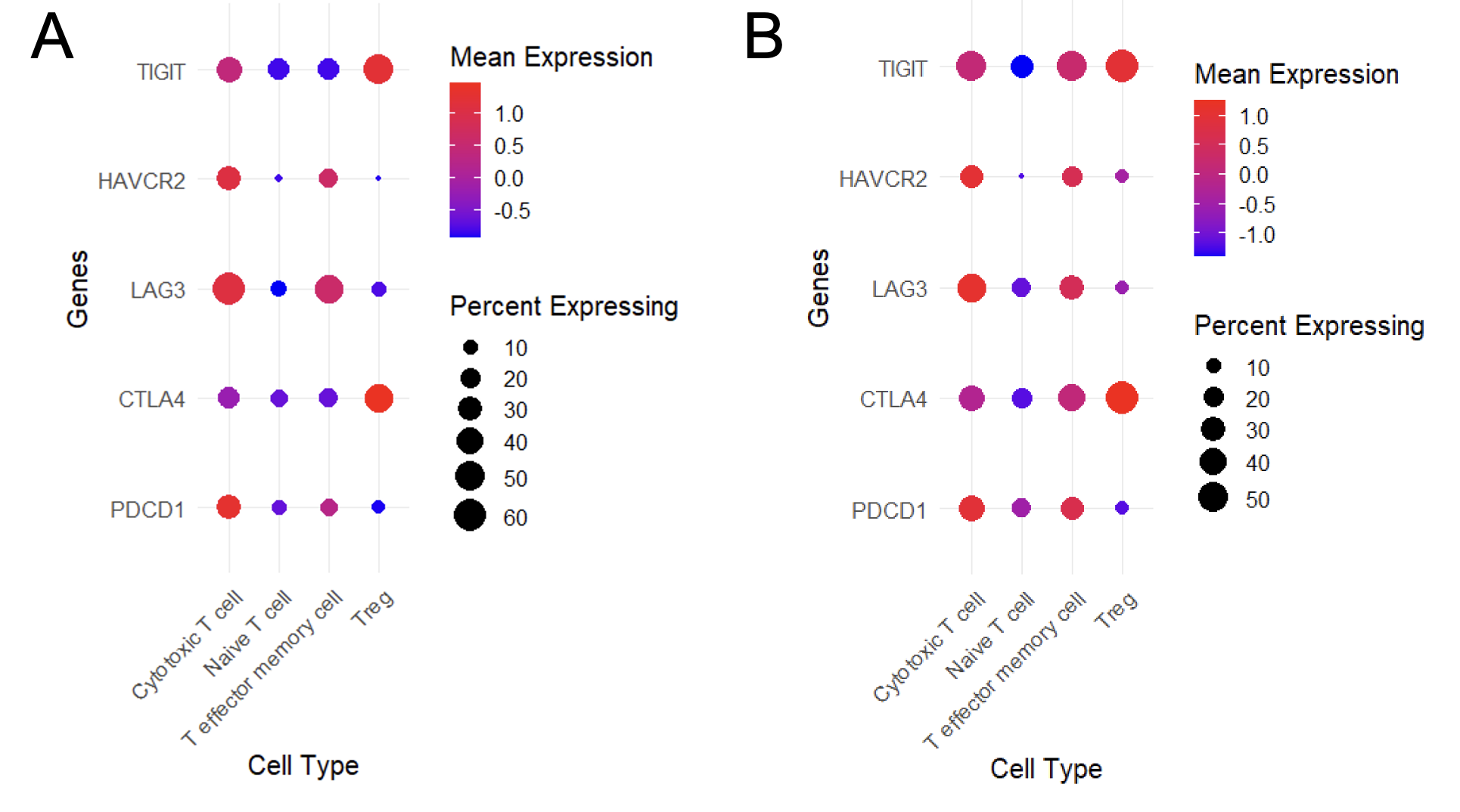
***

***Supplement Figure 6 – Dot plot showing the expression of immune checkpoints across different subtypes of T cells and NK cells detected in UM. A – In primary UM, B – In metastatic UM.***

Mean expression reflects relative expression compared to other cell types on the graph. Expression levels are log-normalized and scaled. The percent expressing indicates how widespread the expression is within each cell type. Treg – regulatory T cells.
